# Intestinal epithelial HDAC3 and MHC class II coordinate microbiota-specific immunity

**DOI:** 10.1172/JCI162190

**Published:** 2023-02-15

**Authors:** Emily M. Eshleman, Tzu-Yu Shao, Vivienne Woo, Taylor Rice, Laura Engleman, Bailey J. Didriksen, Jordan Whitt, David B. Haslam, Sing Sing Way, Theresa Alenghat

**Affiliations:** 1 Division of Immunobiology,; 2 Center for Inflammation and Tolerance,; 3 Division of Infectious Disease, and; 4 Immunology Graduate Program, Cincinnati Children’s Hospital Medical Center and the University of Cincinnati College of Medicine, Cincinnati, Ohio, USA.

**Keywords:** Gastroenterology, Immunology, Adaptive immunity, Antigen presentation, Inflammatory bowel disease

## Abstract

Aberrant immune responses to resident microbes promote inflammatory bowel disease and other chronic inflammatory conditions. However, how microbiota-specific immunity is controlled in mucosal tissues remains poorly understood. Here, we found that mice lacking epithelial expression of microbiota-sensitive histone deacetylase 3 (HDAC3) exhibited increased accumulation of commensal-specific CD4^+^ T cells in the intestine, provoking the hypothesis that epithelial HDAC3 may instruct local microbiota-specific immunity. Consistent with this, microbiota-specific CD4^+^ T cells and epithelial HDAC3 expression were concurrently induced following early-life microbiota colonization. Further, epithelium-intrinsic ablation of HDAC3 decreased commensal-specific Tregs, increased commensal-specific Th17 cells, and promoted T cell–driven colitis. Mechanistically, HDAC3 was essential for NF-κB–dependent regulation of epithelial MHC class II (MHCII). Epithelium-intrinsic MHCII dampened local accumulation of commensal-specific Th17 cells in adult mice and protected against microbiota-triggered inflammation. Remarkably, HDAC3 enabled the microbiota to induce MHCII expression on epithelial cells and limit the number of commensal-specific T cells in the intestine. Collectively, these data reveal a central role for an epithelial histone deacetylase in directing the dynamic balance of tissue-intrinsic CD4^+^ T cell subsets that recognize commensal microbes and control inflammation.

## Graphical abstract

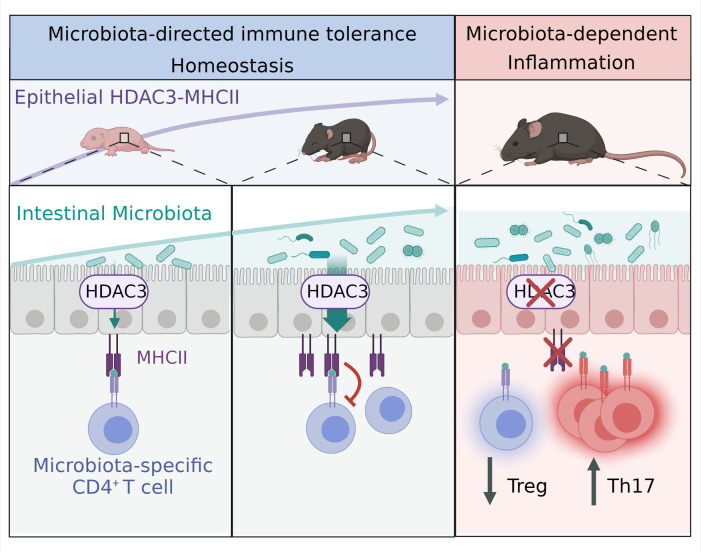

## Introduction

The gastrointestinal tract is home to trillions of microorganisms, collectively termed the microbiota, which form symbiotic relationships with mammalian cells and play a significant role in mediating health and disease. Extensive experiments using broad-spectrum antibiotics or germ-free mouse models have revealed the necessity for the microbiota in the development and function of the host immune system (1–5). Microbiota interactions are particularly impactful during early life, as critical immune education and calibration occur within the first few years of life (6–9). Indeed, disturbances or perturbations in microbiota composition or colonization during this early-life window have been associated with long-lasting changes in immunity that can predispose to the development of chronic inflammatory disorders including asthma, allergy, and inflammatory bowel disease (IBD) (1, 4, 8).

Despite the symbiotic nature of the host-microbiota relationship, the abundance and close association with antigenically foreign microbes at mucosal surfaces pose a potential risk to stimulate pathologic inflammation. This requires that intestinal immune responses must be tightly regulated to allow protective immunity against invading pathogens, while limiting inflammatory responses toward innocuous commensal microbes. Commensal bacteria drive regulatory T cell differentiation (10), and microbiota-reactive effector and memory T cells are present in both mice and humans (11–17). These commensal-specific T cells promote barrier function by inducing protective cytokines and providing cross-reactivity to pathogens (14, 18, 19). However, aberrant immune responses to the microbiota also trigger inflammatory conditions, such as IBD (1, 20–22). In mouse models of colitis, intestinal microbes drive inflammation, in part, by stimulating microbiota-reactive CD4^+^ T cells (23–25). Further, microbiota-specific CD4^+^ T cells in patients with IBD have been shown to be functionally altered and produce more proinflammatory cytokines such as IL-17, compared with T cells from healthy patients (14, 26–30). However, the mechanisms instructing tissue-intrinsic regulation of commensal-specific CD4^+^ T cells remain poorly understood.

Intestinal epithelial cells (IECs) reside at the direct interface between the microbiota and immune cells, and are thus uniquely poised to instruct local immunity in response to microbial antigens. IECs express pathogen recognition receptors that sense microbial signals and regulate intestinal immune responses via secretion of cytokines, chemokines, and growth factors (1, 31). While specialized microfold cells and goblet cell–associated antigen passages in the epithelium deliver antigens to underlying antigen-presenting myeloid cells (32–34), IECs are also equipped with classical antigen processing and presentation pathways that can regulate immune responses (35, 36). However, there remains limited understanding of IEC-directed mechanisms that coordinate healthy microbiota-immune relationships.

Beyond canonical sensors, epithelial expression of the epigenetic-modifying enzyme histone deacetylase 3 (HDAC3) has recently been found to respond to the microbiota and regulate mammalian metabolism, intestinal homeostasis, and inflammation (37–40). Here, we found that microbiota-dependent expansion of commensal-specific CD4^+^ T cells following initial microbiota colonization occurred concurrently with epithelial expression of HDAC3. Given this relationship, we examined whether IEC-intrinsic HDAC3 regulates microbiota-primed CD4^+^ T cell expansion and differentiation. Indeed, loss of HDAC3 expression in IECs resulted in intestinal inflammation and accumulation of microbiota-specific Th17 cells that were directed by epithelial MHCII. Further analysis of tissues from mice reared under germ-free conditions demonstrated that HDAC3 was necessary for microbiota to induce epithelial MHCII–dependent regulation of microbiota-specific CD4^+^ T cells. Taken together, these data reveal that the microbiota induce commensal tolerance and limit inflammation by directing epithelial control of tissue-intrinsic T cells.

## Results

### Epithelial HDAC3 expression limits commensal-specific CD4^+^ T cells in the intestine.

**Figure 1:**
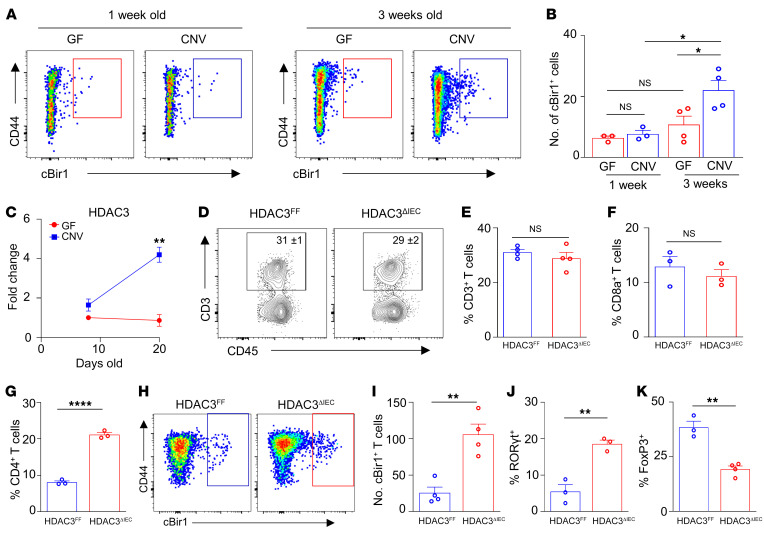
Epithelial HDAC3 expression limits commensal-specific CD4^+^ T cells in the intestine. (**A** and **B**) Number of cBir1^+^ tetramer-specific CD4^+^ T cells isolated from large intestine of neonatal GF and CNV pups. (**C**) mRNA expression of HDAC3 in IECs isolated from large intestine of GF and CNV pups. (**D** and **E**) Frequency of total intestinal CD3^+^. (**F** and **G**) Frequency of total intestinal CD8a^+^ (**F**) and CD4^+^ (**G**) T cells. (**H**–**K**) Number of cBir1^+^ tetramer-specific CD4^+^ T cells (**H** and **I**) and frequency of RORγt^+^ (**J**) and FoxP3^+^ (**K**) cBir1^+^CD4^+^ T cells in large intestine of HDAC3^FF^ and HDAC3^ΔIEC^ mice. cBir1^+^ tetramer cells are gated on live, CD45^+^, lineage (CD11b^–^B220^–^Ly6G^–^, CD11c^–^CD8a^–^, CD4^+^. Data are representative of at least 2 experiments, 3–4 mice per group. *P < 0.05, **P < 0.01, ****P < 0.0001, by 1-way ANOVA with Tukey’s multiple-comparison test (**B**) or unpaired 2-tailed Student’s *t* test (**C**–**K**).

Microbiota colonization begins at birth, and the complexity and density of the microbiota increase most significantly during infancy (41–43). Microbiota exposure beginning in early life promotes immunological education, and disruption during this window can increase chronic inflammatory disorders (42–46). While it has been predicted that initial colonization at birth primes the accumulation of local T cells with specificity to commensal microbes, this has not been directly tested. Therefore, to investigate whether early-life microbiota colonization itself instructs commensal-specific T cell responses in the intestine, CD4^+^ T cells from the large intestine of germ-free (GF) and conventionally housed (CNV) neonatal pups were first analyzed for T cell receptor specificity to the commensal flagella cBir1 peptide using MHCII-restricted tetramers. Within the first week of life, GF and CNV pups exhibited a similarly low abundance of commensal-specific cBir1^+^CD4^+^ T cells in the large intestine (Figure 1, A and B). Comparatively, 3-week-old pups reared in the presence of microbes showed increased accumulation of cBir1^+^ CD4^+^ T cells compared with age-matched GF controls (Figure 1, A and B), which parallels progressively increasing microbiota colonization by weaning (44). Thus, microbiota-derived signals are essential for priming the initial expansion of intestinal microbiota-specific CD4^+^ T cells.

Dysregulated commensal-specific T cells are associated with intestinal inflammation (14), suggesting that impairment in pathways that regulate these T cell subsets can impact susceptibility to pathologic inflammation. Interestingly, IECs from IBD patients express decreased levels of the HDAC3 enzyme (37), and, consistent with findings in other facilities, mice lacking IEC-intrinsic expression of HDAC3 (HDAC3^ΔIEC^ mice) displayed increased susceptibility to chronic intestinal inflammation characterized by rectal prolapse (Supplemental Figure 1A; supplemental material available online with this article; https://doi.org/10.1172/JCI162190DS1), increased levels of the inflammatory biomarker lipocalin-2 (Supplemental Figure 1B), infiltration of inflammatory cells (Supplemental Figure 1, C–E), and histological changes consistent with chronic inflammation (Supplemental Figure 1F) (37). Strikingly, epithelial expression of HDAC3 was also dramatically induced in 3-week-old pups compared with 1-week-old pups (Figure 1C). However, epithelial HDAC3 expression remained at low background levels in these developmental windows for pups raised under GF conditions (Figure 1C), indicating that microbiota colonization increases early-life epithelial HDAC3 expression in the intestine.

The temporal link between epithelium-intrinsic HDAC3 expression and expansion of commensal-specific CD4^+^ T cells following microbiota colonization provoked the hypothesis that IEC-intrinsic HDAC3 may regulate microbiota-specific T cell immunity. In the intestinal lamina propria, total CD3^+^ T cells were unaltered in HDAC3^ΔIEC^ mice compared with Cre-negative littermate HDAC3^FF^ controls (Figure 1, D and E). However, loss of epithelial HDAC3 resulted in elevated intestinal CD4^+^ T cells, but not CD8a^+^ cells (Figure 1, F and G), that exhibited increased cBir1^+^ commensal specificity in the large (Figure 1, H and I) and small intestine (Supplemental Figure 2, A and B). Metagenomic analyses confirmed alterations in the composition of commensal bacteria in the colon of HDAC3^ΔIEC^ mice (Supplemental Figure 3A), characterized by decreased diversity (Supplemental Figure 3B) and reduced Bifidobacteriaceae (Supplemental Figure 3C), in agreement with prior 16S sequencing (37). Further, cBir1 expression itself was decreased in the intestinal microbiota of HDAC3^ΔIEC^ mice relative to littermate controls (Supplemental Figure 3, D and E). Thus, elevated cBir1-commensal-specific T cells in HDAC3^ΔIEC^ mice do not parallel levels of cBir1-expressing species. cBir1^+^ CD4^+^ T cells in HDAC3^ΔIEC^ mice showed increased differentiation into proinflammatory RORγt^+^ Th17 cells (Figure 1J), with reciprocally reduced FoxP3^+^ T regulatory differentiation (Figure 1K) in comparison with floxed controls. Commensal-specific Th1 and T follicular helper (Tfh) cells were unaltered by loss of epithelial HDAC3 (Supplemental Figure 4A). Taken together, these data indicate that epithelial HDAC3 plays a critical role in regulating the balance of commensal-specific Tregs and Th17 cells in the intestine.

### CD4^+^ T cells from mice lacking epithelial HDAC3 induce severe colitis.

**Figure 2:**
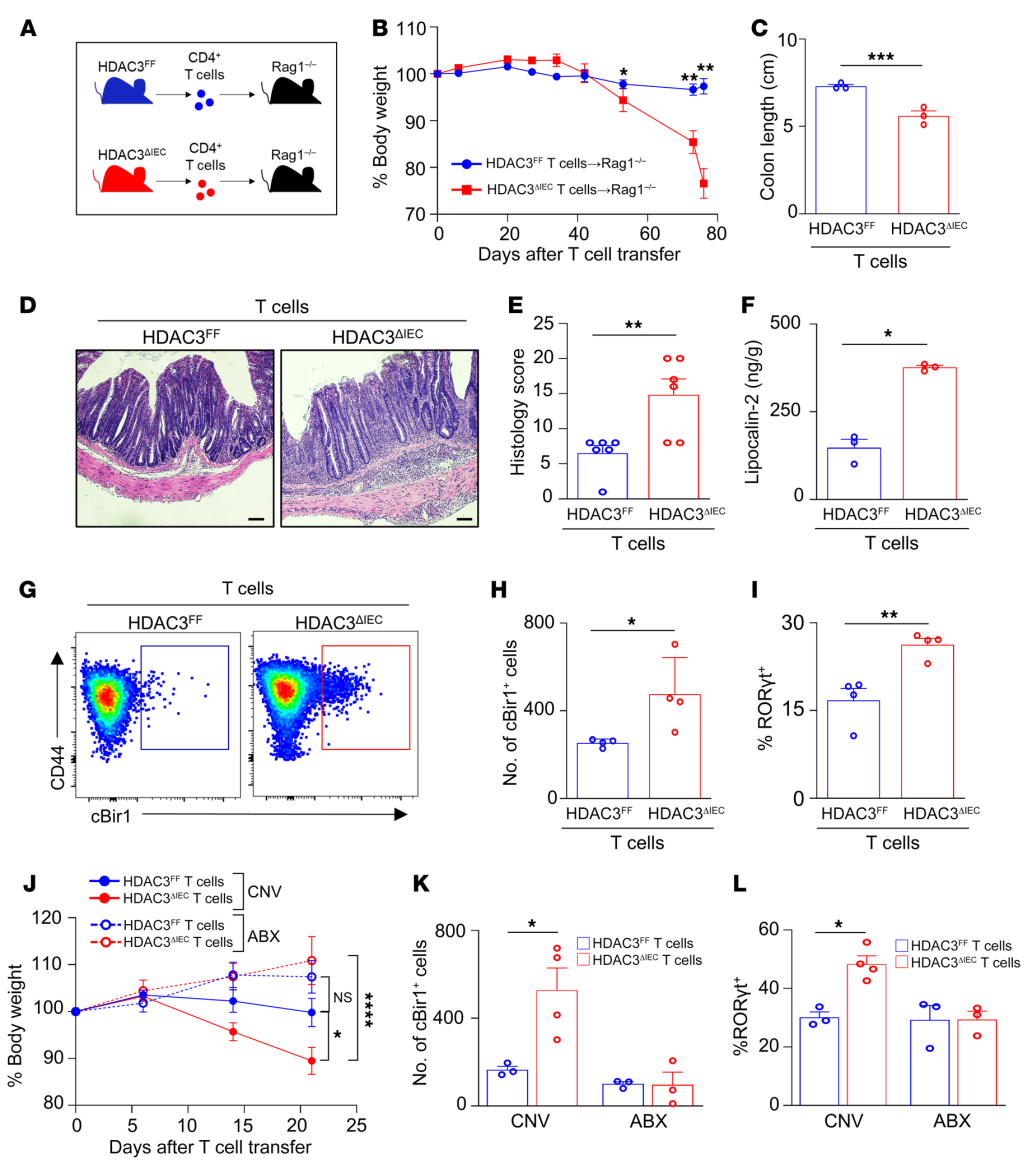
CD4^+^ T cells from mice lacking epithelial HDAC3 induce severe colitis. (**A**) Experimental schematic of T cell colitis model with naive CD4^+^ T cells isolated from HDAC3^FF^ and HDAC3^ΔIEC^ mice transferred into *Rag1^–/–^* hosts. (**B**–**D**) Change in body weight (**B**), colon length (**C**), and H&E-stained colonic sections (**D**) of *Rag1^–/–^* hosts that received T cells from HDAC3^FF^ or HDAC3^ΔIEC^ mice. Scale bars: 20 μM. (**E**) Histological scoring of sections in **D**. (**F**) Fecal concentration of lipocalin-2. (**G** and **H**) Number of cBir1^+^ tetramer-specific CD4^+^ T cells in large intestine of *Rag1^–/–^* hosts that received T cells from HDAC3^FF^ or HDAC3^ΔIEC^ mice. Gated on live, CD45^+^, lineage (CD11b^–^B220^–^Ly6G^–^, CD11c^–^CD8a^–^), CD4^+^. (**I**) Frequency of Th17 (RORγt^+^) of cBir1^+^ CD4^+^ T cells. (**J**–**L**) Change in body weight (**J**), number of cBir1^+^ tetramer-specific CD4^+^ T cells (**K**), and frequency of RORγt^+^ cBir1^+^ CD4^+^ T cells (**L**) in large intestine of *Rag1^–/–^* mice treated with water (CNV) or antibiotics (ABX) that received T cells from HDAC3^FF^ or HDAC3^ΔIEC^ mice. Data are representative of at least 2 independent experiments, 3–4 mice per group. *P < 0.05, **P < 0.01, ***P < 0.001, ****P < 0.0001, by unpaired 2-tailed Student’s *t* test (**B**–**I**) or 1-way ANOVA with Tukey’s multiple-comparison test (**J**–**L**).

To determine whether dysregulated CD4^+^ T cells in HDAC3^ΔIEC^ mice promoted intestinal inflammation, the T cell transfer model of chronic colitis (47–49) was used, in which purified CD4^+^ T cells were isolated from HDAC3^FF^ and HDAC3^ΔIEC^ mice and adoptively transferred into *Rag1^–/–^* recipient mice (Figure 2A). In contrast to *Rag1^–/–^* recipients that received CD4^+^ T cells from control mice, *Rag1^–/–^* recipients receiving CD4^+^ T cells from HDAC3^ΔIEC^ mice displayed more significant weight loss (Figure 2B), colonic shortening (Figure 2C), and severe colitis pathology characterized by inflammatory cell infiltration, crypt hyperplasia, and mural thickening (Figure 2, D and E). Furthermore, luminal concentration of the inflammatory biomarker lipocalin-2 was also significantly upregulated in *Rag1^–/–^* mice that received CD4^+^ T cells from HDAC3^ΔIEC^ mice (Figure 2F). Therefore, IEC-intrinsic HDAC3 expression is essential for suppressing the priming of proinflammatory colitogenic CD4^+^ T cells.

To further investigate colitogenic CD4^+^ T cells isolated from HDAC3^ΔIEC^ mice, their specificity to commensal microbiota–expressed antigens was evaluated. *Rag1^–/–^* recipients that received CD4^+^ T cells from HDAC3^ΔIEC^ mice exhibited elevated microbiota-specific cBir1^+^CD4^+^ T cells compared with hosts that received HDAC3^FF^ cells (Figure 2, G and H). These experiments showed that a majority of cBir1^+^ cells following transfer were RORγt^+^ Th17 cells (Figure 2I and Supplemental Figure 4B). Similar to the skewed differentiation of these cells in HDAC3^ΔIEC^ mice, a higher frequency of commensal-specific T cells differentiated into inflammatory RORγt^+^ Th17 cells when they originated from HDAC3^ΔIEC^ mice compared with HDAC3^FF^ littermate controls (Figure 2I). Commensal-specific T cells from HDAC3^ΔIEC^ mice exhibited reduced differentiation to FoxP3^+^ Tregs, and no difference in Th1 cells (Supplemental Figure 4B). To next test whether these cells were responsive to the microbiota, T cell transfer studies were conducted by depletion of commensal bacteria with broad-spectrum antibiotics (50). Consistent with previous results, increased weight loss (Figure 2J) and expansion of cBir1^+^ Th17 cells were detected in microbiota-replete recipient mice that received T cells from HDAC3^ΔIEC^ mice (Figure 2, K and L). However, colitis-induced weight loss (Figure 2J) and cBir1^+^ Th17 cells were lost with microbiota depletion (Figure 2, K and L), indicating that commensal-specific Th17 cells drive intestinal inflammation in this model. Collectively, these data indicate that epithelial HDAC3 expression is required for regulation of CD4^+^ T cell development in the intestine, as loss of epithelial HDAC3 resulted in increased microbiota-specific colitogenic CD4^+^ T cells.

### HDAC3 regulates surface expression of MHCII on IECs.

The cytokine IFN-γ drives CD4^+^ T cell differentiation into Th1 effectors while suppressing differentiation into other Th lineages, including Th17 cells (51, 52). Previous work demonstrated that during bacterial infection IFN-γ production by intraepithelial T cells was reduced in mice lacking epithelial HDAC3 (39). However, at steady state, IFN-γ levels were low, and significant differences were not detected (Supplemental Figure 4, C and D) (39). In addition, type 1 innate lymphoid cells (ILC1s) can produce IFN-γ. However, loss of epithelial HDAC3 did not affect the frequency of ILC1s, nor the predominant ILC lineage in the colon, ILC3s (Supplemental Figure 4E). Furthermore, commensal-specific Th17 cells remained elevated in HDAC3^ΔIEC^ mice receiving exogenous IFN-γ (Supplemental Figure 4F). Therefore, IFN-γ is unlikely to reflect a direct or primary cause of the basal differences in commensal-specific Th17 cells observed in HDAC3^ΔIEC^ mice.

**Figure 3:**
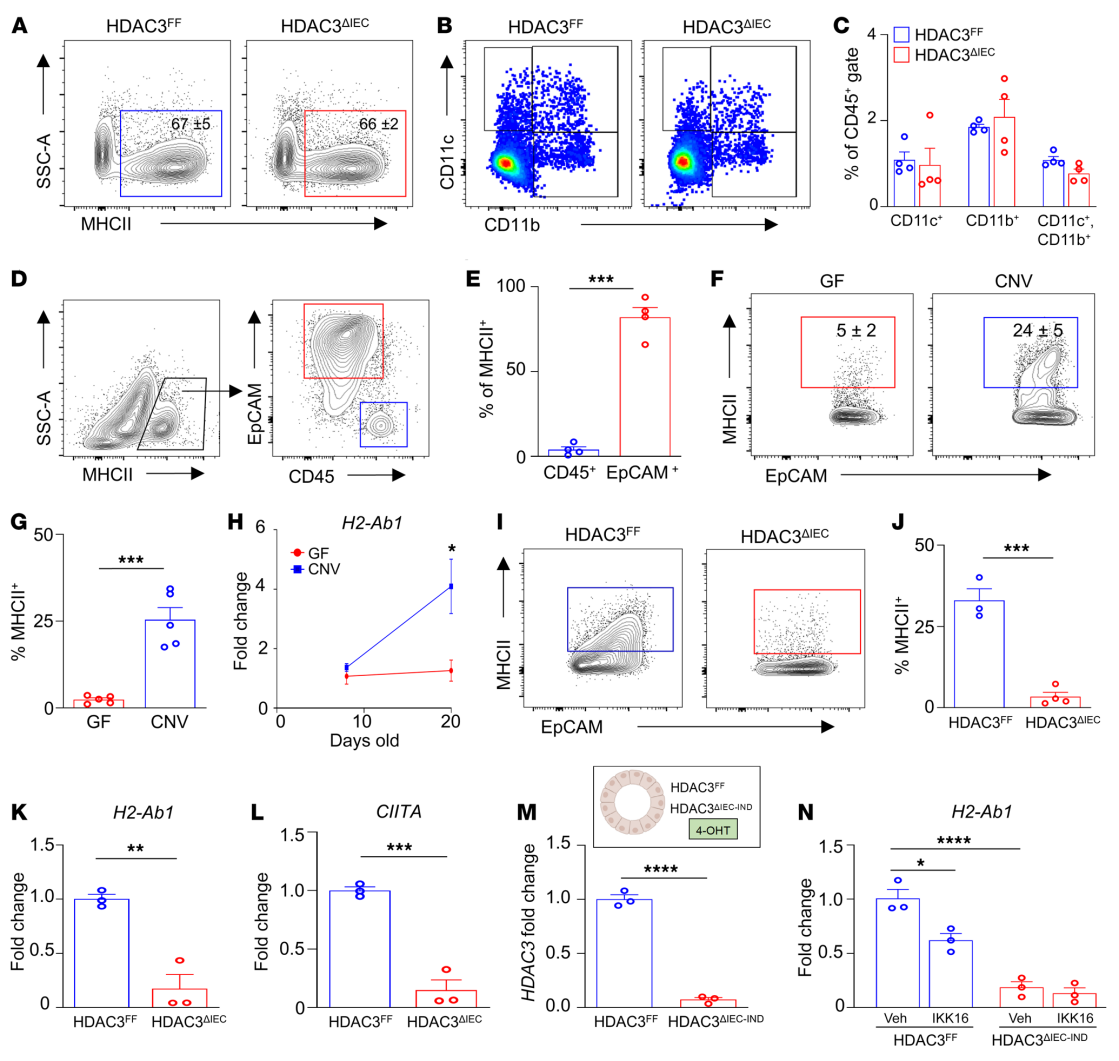
HDAC3 regulates expression of MHCII on IECs. (**A**) Frequency of total MHCII^+^ cells in colon lamina propria. (**B** and **C**) Frequency of dendritic cells and macrophages in large intestinal lamina propria of HDAC3^FF^ and HDAC3^ΔIEC^ mice. Gated on live, CD45^+^, MHCII^+^. (**D**) MHCII^+^ cells at colonic luminal surface. (**E**) Frequency of total MHCII^+^ cells in **D**. (**F** and **G**) Frequency of MHCII^+^ EpCAM^+^ cells in large intestine of GF and CNV mice. (**H**) mRNA expression of H2-Ab1 in IECs isolated from large intestine of GF and CNV pups. (**I** and **J**) Frequency of MHCII^+^ EpCAM^+^ cells in large intestine of HDAC3^FF^ and HDAC3^ΔIEC^ mice. (**K** and **L**) H2-Ab1 (**K**) and CIITA (**L**) mRNA in IECs isolated from the large intestine of HDAC3^FF^ and HDAC3^ΔIEC^ mice. (**M**) HDAC3 mRNA in HDAC3^FF^ and HDAC3^ΔIEC-IND^ organoids treated with tamoxifen (4-OHT). (**N**) H2-Ab1 mRNA expression in HDAC3^FF^ and HDAC3^ΔIEC-IND^ organoids treated with IKK-16. Data are representative of at least 3 independent experiments, 3–5 mice per group. *P < 0.05, **P < 0.01, ***P < 0.001, ****P < 0.0001, by unpaired 2-tailed Student’s *t* test (**E**–**M**) or 1-way ANOVA with Tukey’s multiple-comparison test (**N**).

Antigen presentation via MHCII is critical for instructing antigen-specific CD4^+^ T cell responses. However, the frequency of total MHCII^+^ intestinal hematopoietic cells (Figure 3A) and classical antigen-presenting cells, including MHCII^hi^ intestinal dendritic cells and macrophages, was unaffected by epithelial HDAC3 deletion (Figure 3, B and C). Surprisingly, though, we found that EpCAM^+^ IECs, and not CD45^+^ hematopoietic cells, expressed the majority of MHCII at the intestinal-microbiota interface (Figure 3, D and E). IECs respond to microbial cues, so we next assessed whether the microbiota plays a role in regulating IEC-intrinsic MHCII expression. Consistent with other studies (53–57), IECs isolated from CNV mice displayed higher surface expression of MHCII compared with GF mice (Figure 3, F and G). Further, neonatal GF pups maintained low epithelial expression of *H2-Ab1*, the gene that encodes the β chain of MHCII (Figure 3H). However, epithelial *H2-Ab1* expression was robustly induced following initial microbiota colonization (Figure 3H), similar to regulation of HDAC3 (Figure 1C).

Interestingly, loss of HDAC3 expression dramatically reduced epithelial surface MHCII expression in the large (Figure 3, I and J) and small intestine (Supplemental Figure 5, A and B). IECs from HDAC3^ΔIEC^ mice exhibited reduced *H2-Ab1* gene expression (Figure 3K and Supplemental Figure 5C), suggesting altered transcriptional regulation of the MHCII-encoding gene. Significant changes in histone acetylation did not occur near the *H2-Ab1* gene in IECs from HDAC3^ΔIEC^ mice (Supplemental Figure 5D), suggesting that HDAC3 does not directly target this gene. Expression of the class II coactivator *CIITA* was decreased in IECs from HDAC3^ΔIEC^ mice, relative to IECs from floxed controls (Figure 3L). CIITA is regulated by NF-κB, and HDAC3 has been shown to promote NF-κB activation in multiple cell lineages (58–61). Thus, to test whether NF-κB mediates HDAC3-dependent regulation of MHCII, intestinal organoids were generated from the colon of HDAC3^FF^ and an inducible HDAC3^ΔIEC-IND^ mouse model in which tamoxifen significantly reduces HDAC3 expression (Figure 3M). Inhibition of NF-κB activity suppressed expression of *H2-Ab1* in wild-type organoids (Figure 3N). However, NF-κB inhibition did not impact MHCII expression in organoids that lacked HDAC3 (Figure 3N). Taken together, these data support that HDAC3 regulates epithelial MHCII expression, in part, through activation of NF-κB. As expected, HDAC3^FF^ organoids upregulated *H2-Ab1* expression following IFN-γ stimulation (Supplemental Figure 5E); however, similar induction occurred in organoids lacking HDAC3 expression (Supplemental Figure 5E), supporting that IFN-γ–dependent MHCII regulation remains intact in HDAC3-deficient epithelial cells. While IFN-γ may play a synergistic role in vivo, these organoid data suggest an epithelium-intrinsic NF-κB–dependent mechanism by which HDAC3 controls epithelial MHCII expression.

### Epithelial MHCII limits commensal-specific CD4^+^ T cells and intestinal inflammation.

**Figure 4:**
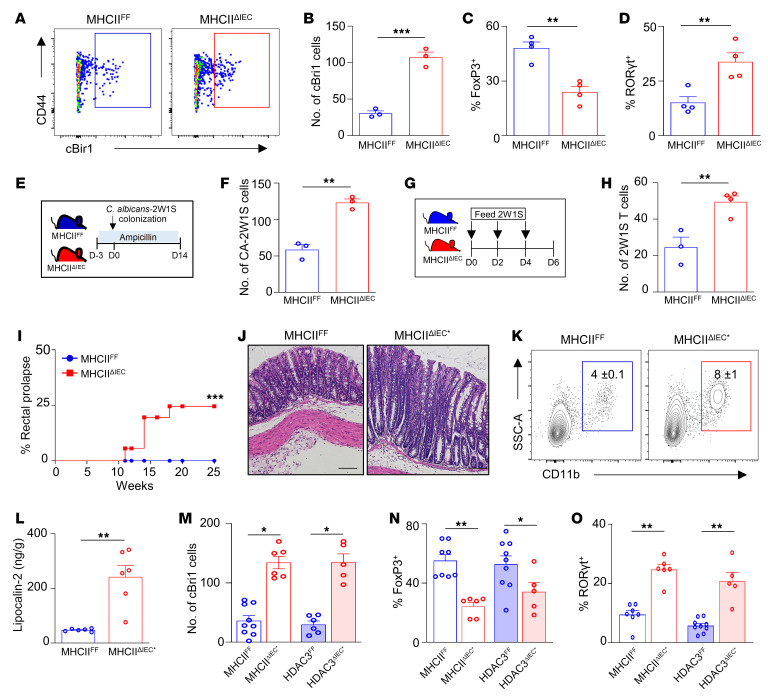
Epithelial MHCII regulates commensal-specific T cells. (**A** and **B**) Number of cBir1^+^-specific CD4^+^ T cells in large intestine of MHCII^FF^ and MHCII^ΔIEC^ mice. (**C** and **D**) Frequency of FoxP3^+^ (**C**) and RORγt^+^ (**D**) cBir1^+^ CD4^+^ T cells in large intestine of MHCII^FF^ and MHCII^ΔIEC^ mice. (**E**) Diagram of 2W1S–*Candida albicans* commensal colonization. (**F**) Number of *C*. *albicans*–2W1S^+^–specific CD4^+^ cells in large intestine of MHCII^FF^ and MHCII^ΔIEC^ mice. (**G**) Diagram of 2W1S peptide feeding model. (**H**) Number of 2W1S^+^-specific CD4^+^ cells in large intestine of MHCII^FF^ and MHCII^ΔIEC^ mice. (**I**) Frequency of rectal prolapse in MHCII^FF^ and MHCII^ΔIEC^ mice. (**J**) H&E-stained colonic sections of MHCII^FF^ and prolapsed MHCII^ΔIEC^ mice (MHCII^ΔIEC*^). Scale bars: 20 μM. (**K** and **L**) Frequency of myeloid cell infiltrate (**K**) and lipocalin-2 levels (**L**) in stool of MHCII^FF^ and MHCII^ΔIEC*^. (**M**–**O**) Number of cBir1^+^-specific CD4^+^ T cells (**M**) and frequency of FoxP3^+^ (**N**) and RORγt^+^ (**O**) cBir1-specific T cells in large intestine of control and prolapsed MHCII^ΔIEC^ and HDAC3^ΔIEC^ mice. Tetramer cells are gated on live, CD45^+^, lineage (CD11b^–^B220^–^Ly6G^–^, CD11c^–^CD8a^–^), CD4^+^. Data are representative of at least 2 independent experiments (**A**–**H**) or are pooled from at least 2 independent experiments (**I**–**O**), 3–6 mice per group. *P < 0.05, **P < 0.01, ***P < 0.001, by unpaired 2-tailed Student’s *t* test (**B**–**H** and **L**–**O**) or Mantel-Cox test (**I**).

While epithelial MHCII has been suggested to function in both protective and detrimental immune responses (53, 57, 62, 63), its role in regulating commensal-specific T cell responses and microbiota-triggered inflammation has remained uncertain. Therefore, to test this, mice with conditional loss of MHCII in IECs (MHCII^ΔIEC^) were generated by crossing of floxed H2-Ab1 mice (MHCII^FF^) with mice expressing Cre recombinase downstream of the villin promoter (64, 65). Significant reduction in MHCII expression in IECs of MHCII^ΔIEC^ mice was confirmed by mRNA (Supplemental Figure 6A) and surface protein analyses (Supplemental Figure 6B). Loss of MHCII expression was restricted to IECs, since levels remained similar on CD45^+^ cells (Supplemental Figure 6C). Interestingly, and in contrast to the activating role of MHCII on classical antigen-presenting cells, we found that loss of IEC-intrinsic MHCII led to elevated accumulation of commensal-specific cBir1^+^CD4^+^ T cells in the colon (Figure 4, A and B) and small intestine (Supplemental Figure 6D). Intestine from MHCII^ΔIEC^ mice exhibited reduced commensal-specific Tregs (Figure 4C) and an increase in commensal-specific Th17 cells (Figure 4D), whereas microbiota-specific Th1 and Tfh cells were similar (Supplemental Figure 6E). Importantly, the composition and diversity of the intestinal microbiota were similar in MHCII^ΔIEC^ mice and floxed littermate controls (Supplemental Figure 7, A–C), including the proportion of bacteria harboring the cBir1 gene (Supplemental Figure 7, D and E). Thus, elevated cBir1 commensal–specific T cells in the intestine of MHCII^ΔIEC^ mice do not reflect alterations in the composition of commensal bacteria.

In order to examine whether this commensal-specific response occurred with another microbial antigen, mice were also colonized with *Candida albicans* expressing the 2W1S_55–68_ variant of I-Eα epitope (Figure 4E) (16, 17). Consistent with regulation of cBir1^+^CD4^+^ T cells, MHCII^ΔIEC^ mice displayed increased numbers of *C.* *albicans*–2W1S–specific CD4^+^ T cells relative to MHCII^FF^ mice (Figure 4F) with similar levels of *C. albicans* colonization. Further, epithelial MHCII was required to limit intestinal accumulation of 2W1S^+^CD4^+^ T cells following oral administration of the 2W1S peptide (Figure 4, G and H) (66). To investigate the mechanism by which epithelial MHCII regulates tissue-intrinsic T cells, the proliferation, survival, and anergy of microbiota-specific T cells were compared in MHCII^FF^ and MHCII^ΔIEC^ mice. Loss of epithelial MHCII led to minimal differences in the proliferation marker Ki67 (Supplemental Figure 8A) and the anergy markers FR4 and CD73 (Supplemental Figure 8, B and C). Surprisingly, cBir1^+^CD4^+^ T cells from MHCII^ΔIEC^ mice exhibited a reduction in the apoptosis marker Bim (Supplemental Figure 8, D and E) and annexin V (Supplemental Figure 8F), indicating that epithelial MHCII may promote apoptosis, leading to their local accumulation in MHCII^ΔIEC^ mice.

Mice lacking IEC-intrinsic HDAC3 exhibited increased susceptibility to chronic intestinal inflammation (Supplemental Figure 1). Remarkably, similarly to HDAC3^ΔIEC^ mice, mice lacking epithelial MHCII also displayed an increased incidence of rectal prolapse with age indicative of intestinal inflammation (Figure 4I). Furthermore, prolapsed MHCII^ΔIEC^ mice (MHCII^ΔIEC*^) demonstrated increased pathology consistent with chronic colitis (Figure 4J), increased infiltration of myeloid cells (Figure 4K), and elevated fecal lipocalin-2 (Figure 4L). Interestingly, MHCII^ΔIEC^ mice and HDAC3^ΔIEC^ mice with an increased propensity to prolapse were characterized by elevated levels of commensal-specific CD4^+^ T cells (Figure 4M) that reflected a reduction in microbiota-specific FoxP3^+^ Tregs (Figure 4N) and an increase in commensal-specific RORγt^+^ inflammatory Th17 cells (Figure 4O). Collectively, these data suggest that epithelial HDAC3 may be critical in regulating microbiota-induced MHCII-directed commensal-specific immunity and inflammation.

### HDAC3 enables microbiota to regulate epithelial-dependent commensal-specific immunity.

**Figure 5:**
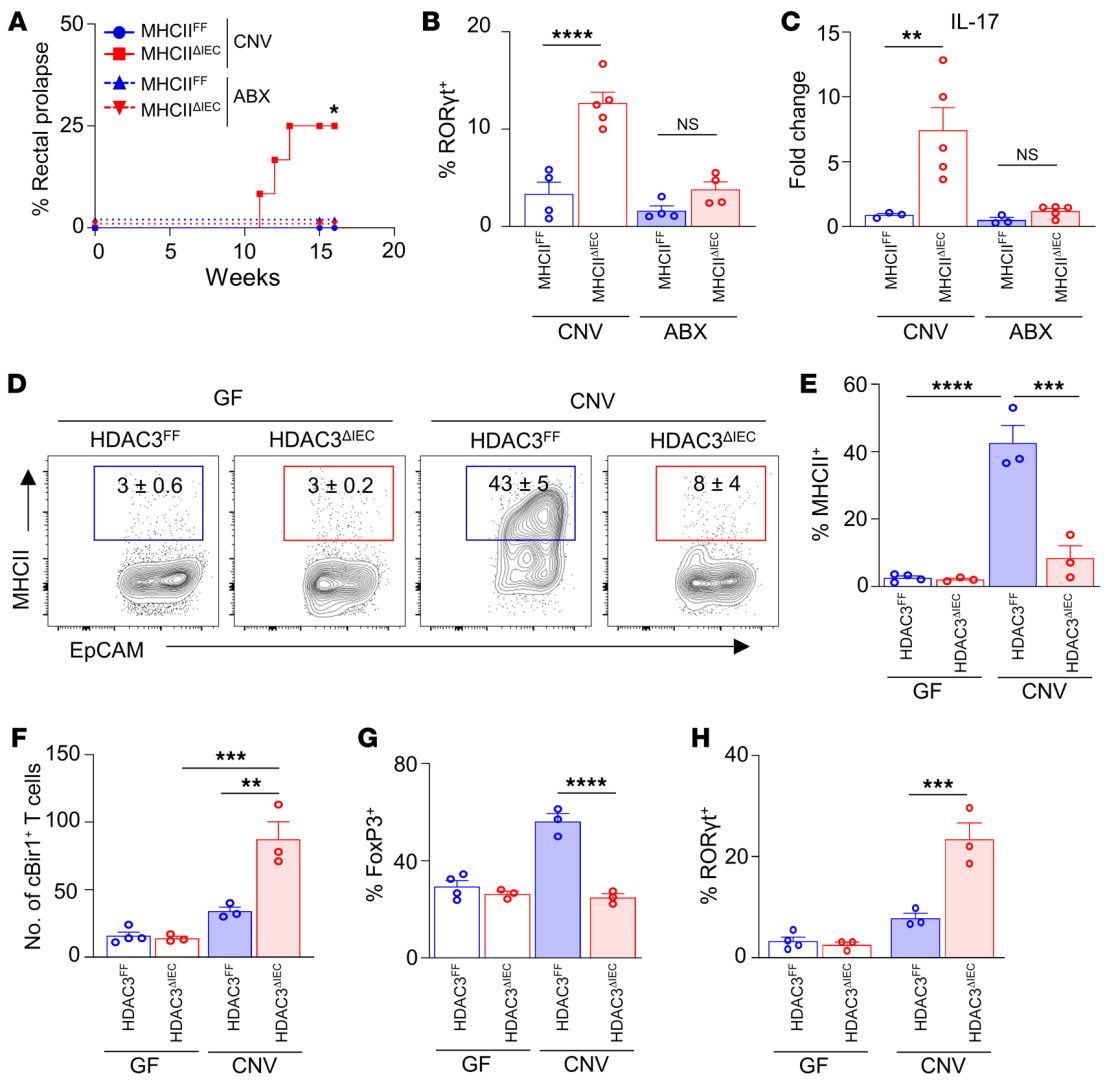
HDAC3 enables microbiota to regulate epithelium-dependent, commensal-specific immunity. (**A**) Frequency of rectal prolapse in control (CNV) and antibiotic-treated (ABX) MHCII^FF^ and MHCII^ΔIEC^ mice. (**B** and **C**) Frequency of RORγt^+^ cBir1^+^-specific CD4^+^ T cells (**B**) and IL-17 mRNA expression (**C**) in large intestine of control and ABX-treated MHCII^FF^ and MHCII^ΔIEC^ mice. (**D** and **E**) Frequency of MHCII^+^ EpCAM^+^ cells in large intestine of GF- and CNV- HDAC3^FF^ and HDAC3^ΔIEC^ mice. (**F**–**H**) Number of cBir1^+^ tetramer-specific CD4^+^ T cells (**F**) and frequency of FoxP3^+^ (**G**) and RORγt^+^ (**H**) cBir1^+^ T cells isolated from GF- and CNV-HDAC3^FF^ and HDAC3^ΔIEC^ mice. cBir1^+^ tetramer cells are gated on live, CD45^+^, lineage (CD11b^–^B220^–^Ly6G^–^, CD11c^–^CD8a^–^), CD4^+^. Data are representative of at least 2 independent experiments, 3–5 mice per group. *P < 0.05, **P < 0.01, ***P < 0.001, ****P < 0.0001, by Mantel-Cox (**A**) or 1-way ANOVA with Tukey’s multiple-comparison test (**B**–**H**).

To test the requirement for the microbiota in MHCII-dependent intestinal inflammation and commensal-specific immunity, the drinking water of MHCII^ΔIEC^ and control MHCII^FF^ mice was supplemented with broad-spectrum antibiotics that significantly deplete commensal bacteria (50). Consistent with earlier results (Figure 4I), MHCII^ΔIEC^ mice exhibited an increased prevalence of rectal prolapse (Figure 5A), whereas depletion of the microbiota prevented spontaneous intestinal inflammation in MHCII^ΔIEC^ mice (Figure 5A). Furthermore, microbiota-replete MHCII^ΔIEC^ mice exhibited accumulation of commensal-specific Th17 cells (Figure 5B) and increased levels of IL-17 (Figure 5C). However, antibiotic-treated MHCII^ΔIEC^ mice did not demonstrate increased commensal-specific Th17 cells (Figure 5B) or IL-17 expression (Figure 5C) relative to MHCII^FF^ mice. Taken together, these data highlight the necessity for the microbiota in triggering epithelial MHCII–dependent intestinal inflammation and commensal-specific immune responses.

To test whether HDAC3 is necessary in mediating this regulation of epithelial and immune cells by the microbiota, GF-derived HDAC3^FF^ and HDAC3^ΔIEC^ mice were compared with CNV-HDAC3^FF^ and -HDAC3^ΔIEC^ mice. Consistent with earlier data using wild-type CNV and GF mice (Figure 3, F and G), IEC-intrinsic MHCII expression was significantly induced in CNV-HDAC3^FF^ controls compared with GF-HDAC3^FF^ mice (Figure 5, D and E), whereas CNV-HDAC3^ΔIEC^ mice failed to upregulate MHCII (Figure 5, D and E). In GF mice, loss of epithelial HDAC3 expression had no impact on protein levels of MHCII on IECs (Figure 5, D and E), demonstrating the specific necessity for HDAC3 in mediating microbiota-dependent regulation of epithelial MHCII. Furthermore, CNV-HDAC3^ΔIEC^ mice exhibited reduced cBir1^+^ commensal-specific FoxP3^+^ and elevated commensal-specific Th17 cells as compared with CNV-HDAC3^FF^ littermate controls (Figure 5, F–H). However, GF-HDAC3^ΔIEC^ had levels of commensal-specific Tregs and Th17 cells similar to those of GF-HDAC3^FF^ mice (Figure 5, F–H). These data indicate the necessity for HDAC3 in enabling the microbiota to regulate commensal-specific immunity. Collectively, these findings reveal that microbiota colonization can induce commensal self-tolerance through an epithelial HDAC3/MHCII regulatory pathway that dampens commensal-specific immune responses directly in the local tissue environment (Supplemental Figure 9).

## Discussion

The microbiota is essential for the development and education of the host immune system. However, inappropriate immune reactions to the microbiota underlie several chronic inflammatory conditions, highlighting the necessity to understand how microbiota-specific immunity is controlled. In this study, we found that initial microbiota colonization led to upregulation of epithelial HDAC3 expression and expansion of commensal-specific T cells. Selective deletion of HDAC3 in IECs resulted in decreased epithelial MHCII expression and impaired regulation of commensal-specific CD4^+^ T cells that are controlled by epithelial MHCII. Importantly, loss of epithelial expression of HDAC3 or MHCII resulted in reduced commensal-specific Tregs, concurrent with the increase in commensal-specific Th17 cells. These results align with recent human data suggesting that commensal-specific T cells switch from tolerogenic cells in healthy individuals to inflammatory IL-17–secreting cells in patients with active Crohn’s disease (14, 67). While the composition of commensal bacteria differs in HDAC3^ΔIEC^ versus MHCII^ΔIEC^ mouse models, both models exhibited a similar increase in cBir1^+^ commensal-specific CD4^+^ T cells. Consistent with host-intrinsic regulation of microbiota-specific T cells, HDAC3 was necessary for microbiota to induce epithelial MHCII–dependent regulation of microbiota-specific T cells. Therefore, commensal microbes can direct self-tolerance by inducing an HDAC3-dependent epithelial MHCII pathway that regulates local dynamics of commensal-specific CD4^+^ T cell subsets and susceptibility to microbiota-sensitive disease (Supplemental Figure 9).

Microbiota-specific CD4^+^ T cells are generated centrally in the thymus (68) and in peripheral mucosal tissues (15, 69–71). While autoreactive CD4^+^ T cells are well known to be limited by thymic negative selection (72–74), the mechanisms controlling commensal-specific cells remain less well understood. Previous work described that negative selection of activated commensal-specific T cells can be mediated by MHCII-expressing group 3 innate lymphoid cells (ILC3s) (75, 76). While ILC3s are relatively rare cells at the luminal surface of the intestine, we show that IECs are a major source of intestinal MHCII. HDAC3-dependent MHCII expression specifically by epithelial cells limited accumulation of commensal-specific Th17 and, thus, may be a dominant mechanism at the luminal surface for controlling commensal-specific CD4^+^ T cells and dampening pathogenic responses to the microbiota. In addition to controlling commensal-specific T cell accumulation, loss of epithelial HDAC3 or MHCII resulted in a significant reduction of commensal-specific Tregs, similar to recent observations with RORγt^+^ antigen-presenting cells (77–79). However, it is likely that CD4^+^ T cell responses to the microbiota reflect multiple signaling pathways in vivo that are not limited to HDAC3. Thus, complementary and essential roles for antigen presentation pathways in distinct nonclassical cells seem to be crucial for establishing and maintaining healthy intestine-intrinsic tissue homeostasis.

Studies on epithelial MHCII in the control of intestinal health and disease have led to varied or conflicting results, highlighting the context-dependent role of this regulation. Previous studies have suggested that loss of epithelial MHCII protected mice subjected to models of T cell–driven intestinal inflammation and graft-versus-host disease (53, 62). In contrast, other reports have shown that epithelial MHCII promotes bulk Treg development and IL-10 expression by CD4^+^ T cells (57, 80) and limited CD4^+^ T cell activation (81). Furthermore, IECs express limited costimulatory molecules (82–84), suggesting that epithelial MHCII expression may promote tolerogenic T cell responses, unlike classical antigen-presenting cells. Our data in fact align with predictions from these latter studies that epithelial MHCII promotes intestinal homeostasis by controlling commensal-specific T cell responses. Indeed, loss of epithelial HDAC3-dependent MHCII results in a reduction of commensal-specific Tregs with a concurrent increase in Th17 cells. Consistent with predictions by Tuganbaev et al. (57), our 2W1S data also support that epithelial MHCII may regulate intestinal immune responses directed toward ingested antigens. Thus, epithelial MHCII–expressing cells potentially direct broader regulation of immune tolerance to both resident microbes and food antigens to control pathologic inflammation in the intestine.

Loss of HDAC3-dependent epithelial MHCII led to elevated commensal-specific Th17 activity and increased susceptibility to microbiota-driven intestinal inflammation (Supplemental Figure 9). IFN-γ amplifies Th1 responses while inhibiting the differentiation and function of other Th subsets, including Th17 cells (51, 52). Moreover, IL-17 can also suppress the production of IFN-γ while limiting Th1 differentiation (85). However, in our system IFN-γ is not a primary factor driving the Th17 phenotype observed in HDAC3^ΔIEC^ mice. In addition, elevated commensal-specific Th17 cells were also found in mice with loss of epithelial MHCII. Remarkably, patients with active IBD have functionally distinct microbiota-reactive CD4^+^ T cells, compared with those found in healthy controls, and secrete higher levels of IL-17 (14, 26, 28–30, 67). IL-17 has been linked to development and exacerbation of several autoimmune and inflammatory conditions, including IBD (28, 30, 86). Furthermore, mouse models with disrupted IL-17 signaling are protected from intestinal inflammation (87, 88), supporting a pathogenic role for IL-17 in intestinal inflammation. In addition, microbiota-specific Th17 cells are required for driving T cell–dependent colitis (89), and transfer of RORγt- or IL-17–deficient T cells protects from the development of intestinal inflammation (90). While it is not feasible to directly transfer endogenous commensal-specific T cells owing to the rarity of cells positive for specific tetramers, expansion of cBir1^+^ Th17 cells required the presence of the microbiota, suggesting that commensal-reactive Th17s are crucial for inducing intestinal inflammation. However, clinical trials depleting IL-17 in IBD patients have been ineffective, and in some cases worsened disease (91). This pleiotropic role for IL-17 in regulating intestinal homeostasis suggests that targeting specific IL-17 producers, instead of broadly neutralizing IL-17 itself, may be a more effective treatment. In fact, work has shown that therapeutics that target commensal-specific Th17 cells, while retaining IL-17 production from other cell types, reduced intestinal inflammation (92). Our data suggest that promoting epithelial MHCII expression through enhanced HDAC3 activity may further restrict proinflammatory commensal-specific T cells and promote healthy intestinal homeostasis.

Crosstalk between the microbiota and mammalian immune cells involves communication via pattern recognition receptor engagement and microbiota-derived metabolite signaling. Our data reveal a distinct level of epithelial regulation in which the microbiota-sensitive enzyme HDAC3 regulates epithelial MHCII expression in the intestine. Indeed, loss of epithelial HDAC3 was sufficient to abrogate microbiota-dependent MHCII expression in the intestine. Previous studies have shown that the microbiota are required for IEC-intrinsic MHCII expression (53–57). Many of these studies have focused on how immune cells can induce epithelial MHCII through IFN-γ signaling (53, 93, 94). IFN-γ amounts used for in vitro studies are relatively high compared with basal homeostatic concentrations in the intestine. However, despite this limitation, our work suggests an additional epithelial cell–intrinsic mechanism through which the microbiota promotes epithelial MHCII expression via HDAC3. In addition, disruption to canonical pattern recognition pathways leads to loss of intestinal barrier integrity and increased inflammation (95–97), and the TLR signaling adaptors MyD88 and TRAF have been shown to induce epithelial MHCII expression, particularly in the small intestine (53). MyD88/TRAF signaling promotes NF-κB activation, which can drive CIITA and MHCII, whereas HDAC3 has been found to regulate NF-κB activation (58, 59, 61, 98, 99). Our data indicate that NF-κB induces epithelial MHCII expression in an HDAC3-dependent manner. HDAC3 is a multifaceted enzyme that can deacetylate histone and nonhistone targets to alter gene expression and cellular functions. Thus, transcriptional differences observed in HDAC3^ΔIEC^ mice represent a collective outcome resulting from altered regulation of histones, and potentially nonhistone targets and enzyme-independent roles (59, 60, 100–103). Despite this IEC-intrinsic mechanism, we cannot exclude a synergistic role for IFN-γ in promoting epithelial MHCII or that HDAC3-dependent regulation of MHCII in vivo reflects integrated networks of cell-intrinsic and -extrinsic pathways.

Increasing evidence suggests that several tissue-specific, non-hematopoietic cells, including lung epithelial cells, skin keratinocytes, and fibroblasts, express MHCII and the necessary machinery to regulate tissue-intrinsic CD4^+^ T cell responses (104–106). Given the ubiquitous nature of HDAC3, it is thus plausible that HDAC3 promotes MHCII-dependent pathways in other nonclassical antigen-presenting cells as well. Interestingly, tissue-specific deletion of HDAC3 has been associated with chronic inflammatory disease models for diabetes, heart disease, Alzheimer’s disease, and IBD (37, 38, 107–109). Importantly, our findings here uncover a fundamental new tenet for immune regulation whereby HDAC3 induction of nonhematopoietic epithelial MHCII is largely induced by the microbiota to dampen local self-directed immune responses. These findings reveal a central host mechanism that is utilized by the microbiota to instruct commensal-directed immunity, and suggest that this regulatory pathway can be targeted to treat chronic inflammatory conditions.

## Methods

### Mice.

C57BL/6J mice were purchased from The Jackson Laboratory and maintained in our specific pathogen–free conventional (CNV) facility at Cincinnati Children’s Hospital Medical Center (CCHMC). Germ-free (GF) mice were maintained in flexible isolators in the CCHMC Gnotobiotic Mouse Facility, fed autoclaved feed and water, and monitored for absence of microbes. HDAC3^FF^, HDAC3^ΔIEC^, and HDAC3^ΔIEC-IND^ mice were generated as previously described (37). H2-Ab1(MHCII)^FF^ and *Rag1^–/–^* mice were purchased from The Jackson Laboratory and maintained at CCHMC. MHCII^FF^ mice were crossed to C57BL/6J-Villin-Cre to generate MHCII^ΔIEC^ mice. Sex- and age-matched littermate controls were used for all studies. Mice were housed up to 4 per cage in a ventilated cage system on a 12-hour light/12-hour dark cycle, with free access to water and food. All mouse studies were conducted with approval by the Institutional Animal Care and Use Committee at CCHMC. These protocols follow standards enacted by the US Public Health Services and Department of Agriculture. All experiments followed standards set forth by Animal Research: Reporting of In Vivo Experiments (ARRIVE).

### Murine colitis and 2W1S models.

For the T cell transfer colitis model, 5 × 10^5^ naive CD4^+^ T cells were isolated from the spleen and mesenteric lymph nodes of HDAC3^FF^ and HDAC3^ΔIEC^ mice via MojoSort Mouse CD4 Naive T Cell Isolation (BioLegend). Cell purity was confirmed by flow cytometry and T cells injected i.p. into age- and sex-matched *Rag1^–/–^* recipients. For antibiotic treatment, MHCII^FF^ and MHCII^ΔIEC^ mouse pups were provided with water supplemented with 1 mg/mL colistin (MilliporeSigma), 1 mg/mL ampicillin (MilliporeSigma), and 5 mg/mL streptomycin (MilliporeSigma) at weaning and maintained on antibiotics for 16 weeks. *Rag1^–/–^* mice were provided with the same antibiotic cocktail for 7–10 days before T cell transfer, then maintained on antibiotic-water for the duration of the experiment. Antibiotic-water was refreshed every 7–10 days. Mice receiving PBS or 10 μg of recombinant IFN-γ (PeproTech) i.p. were analyzed after 24 hours. *C.* *albicans*–2W1S colonization was conducted as previously described (16, 17, 110). Briefly, mice were pretreated with ampicillin-water (1 mg/mL) 2 days before colonization and maintained on ampicillin-water for the duration of the experiment. Mice were given recombinant *C*. *albicans* expressing 2W1S_55–68_ peptide dropwise into the mouth (111). Mice were monitored for colonization by plating of fecal CFUs, harvested 14 days after colonization. For feeding of 2W1S, MHCII^FF^ and MHCII^ΔIEC^ mice were orally gavaged with 100 μg of 2W1S peptide on day 0, day 2, and day 4, and then 2W1S-specific cells were harvested on day 6 as previously described (66).

### Cell isolation.

The large intestine was harvested, opened, and washed in PBS. For IECs, tissue was placed in pre-warmed strip buffer (PBS, 5% FBS, 1 mM EDTA, 1 mM DTT) and incubated at 37°C at a 45° angle with shaking at 180 rpm for 15 minutes. For lamina propria isolation, tissue was washed with PBS to remove EDTA and DTT, then incubated in pre-warmed digestion buffer (RPMI with 1 mg/mL Collagenase/Dispase [MilliporeSigma]) at 37°C at a 45° angle with shaking at 180 rpm for 30 minutes. After incubation, the tissue was vortexed and passed through a 70 μm cell strainer.

### Flow cytometry.

Cells were stained for flow cytometry using the following antibodies diluted in FACS buffer (2% FBS, 0.01% sodium azide, PBS): BV711–anti-CD326 (EpCAM) (clone G8.8, BioLegend), BUV395–anti-CD45.2 (clone 104, BD Biosciences), APC– or FITC–anti-MHCII (clone M5/114.15.2, eBioscience), APC–eFluor 780–anti-CD4 (clone RM4-5, eBioscience), PE-Cy7–anti-CD44 (clone IM7, eBioscience), Alexa Fluor 647– or BV650–anti-RORγt (clone Q31-378, BD Biosciences), PerCP–eFluor 710– or APC–anti-CD8a (clone 53-6.7, eBioscience), PerCP-Cy5.5–anti-CD3 (clone 17A2, eBioscience), PerCP–eFluor 710–anti-B220 (clone RA3-6B2, eBioscience), PerCP–eFluor 710–anti-Ly6G (clone 1A8-Ly6g, eBioscience), PerCP-Cy5.5–, eFluor 450–, or PE–anti-CD11b (clone M1/70, eBioscience), PerCP-Cy5.5–anti-CD11c (clone N418, eBioscience), BV650–anti-CXCR5 (clone 138D7, BioLegend), Alexa Fluor 647–anti-FR4 (clone 12A5, BioLegend), eFluor 450–anti-CD73 (clone eBioTY/11.8, eBioscience), Alexa Fluor 488–anti-Bim (C34C5, Cell Signaling Technology), BV421–anti-Tbet (clone 4B10, BioLegend), FITC–anti-CD90.2 (clone 53-2.1, eBioscience), PE-Cy7–anti-CD127 (clone A7R34, eBioscience), eFluor 450–anti-Ki67 (clone SolA15, eBioscience), and PE–anti–IFN-γ (clone XMG1.2, eBioscience). For cytokine staining, cells were stimulated with 50 ng of PMA and 1 μg of ionomycin for 3–4 hours at 37°C. Cells were stained with Alexa Fluor 488– or APC-conjugated annexin V (eBioscience) diluted in Annexin V staining buffer (BD Pharmingen). Dead cells were excluded with the Fixable Aqua Dead Cell Stain Kit (Invitrogen). The BD Fix/Perm kit was used for intracellular staining. Class II–restricted tetramers (cBir1: YSNANILSQ; and 2W1S: EAWGALANWAVDSA) were PE conjugated and were generated and provided by the NIH tetramer core. For tetramer staining, samples were incubated with tetramers (1:100) and Fc Block (anti–mouse CD16/CD32, eBioscience) for 1 hour at room temperature. Samples were acquired on the BD LSRFortessa and analyzed with FlowJo Software (Tree Star).

### RNA and quantitative PCR analysis.

RNA was isolated using the RNeasy Kit (Qiagen). For RNA from whole tissue, samples were homogenized in TRIzol. Chloroform was added for phase separation, and RNA was precipitated by mixing with isopropanol. cDNA was synthesized using the Verso reverse transcriptase kit (Thermo Fisher Scientific) following the manufacturer’s protocol. Real-time PCR was performed using SYBR Green (Applied Biosystems) and analyzed using the following murine primer sequences: HPRT forward 5′-GATTAGCGATGAACCAGGT-3′, HPRT reverse 5′-CCTCCCATCTCCTTCATGACA-3′, H2-Ab1 forward 5′-TGTGAGTCCTGGTGACTGCCATTA-3′, H2-Ab1 reverse 5′-TCGCCCATGAACTGGTACACGAAA-3′, IL-17 forward 5′-ACCGCAATGAAGACCCTGAT-3′, IL-17 reverse 5′-TCCCTCCGCATTGACACA-3′, HDAC3 forward 5′-TTGGTATCCTGGAGCTGCTT-3′, HDAC3 reverse 5′-GACCCGGTCAGTGAGGTAGA-3′, CIITA forward 5′-CCCTGCGTGTGATGGATGTC-3′, CIITA reverse 5′-ATCTCAGACTGATCCTGGCAT-3′.

### Lipocalin-2 ELISA.

Fecal pellets were homogenized in PBS at a concentration of 100 mg/mL, then centrifuged at high speed for 10 minutes. Supernatants were collected, and lipocalin-2 levels were determined using a mouse Lipocalin-2/NGAL ELISA kit (R&D Systems) following the manufacturer’s instructions.

### Intestinal organoids.

Intestinal organoids were generated from the colon of HDAC3^FF^ and HDAC3^ΔIEC-IND^ mice as previously described (38, 112). Briefly, the colon was opened, cut into small pieces, and incubated in chelation buffer (2 mM EDTA in PBS) for 30 minutes at 4°C with rotation. Tissue was then transferred to shaking buffer (PBS, 43.3 mM sucrose, 54.9 mM sorbitol) and shaken by hand for 2–5 minutes. Colonic crypts were resuspended and plated in Matrigel (Corning) with organoid culture medium (60% Advanced DMEM/F12 medium [Gibco, Thermo Fisher Scientific] supplemented with 10 mM HEPES, 2 mM l-glutamate, 40% L-WRN conditioned medium, 1× N2 supplement, 1× B27 supplement, 50 ng/mL murine EGF, and 10 μM Y-27632 ROCK inhibitor). Culture medium was refreshed every 2–3 days. To induce HDAC3 deletion, organoids were treated with 1 μM hydroxytamoxifen (4-OHT, MilliporeSigma) for 24 hours. Organoids were treated with vehicle (DMSO) or 5 μM IKK-16 (Selleckchem) for 24 hours. For IFN-γ stimulation, organoids were treated with 100 U/mL of mouse recombinant IFN-γ (BioLegend) for 24 hours. After incubation, organoids were washed 3 times in PBS and lysed using the RNeasy kit (Qiagen).

### Microbiota analyses.

For shotgun metagenome sequencing, DNA was extracted from approximately 0.1 g of stool using the PowerFecal DNA isolation kit (Qiagen Inc.) per manufacturer recommendations. Sequencing libraries were generated from microbial DNA using the Nextera XT protocol (Illumina). Sequencing was performed on an Illumina NovaSeq 6000 machine using 150-bp DNA paired-end reads to a depth of approximately 4 G base pairs per sample. Raw sequence data were de-multiplexed and converted to FASTA format and subjected to downstream analysis. Paired-end sequencing reads from each sample were aligned with Kraken (113) against a custom genome database consisting of the human genome and approximately 40,054 bacterial, fungal, viral, and parasitic genomes. The database was derived initially from all bacteria, fungi, and viruses in the RefSeq genome database (https://www.ncbi.nlm.nih.gov/refseq/) as well as the human genome (GR38Ch; https://www.ncbi.nlm.nih.gov/projects/genome/guide/human/index.shtml). Manual curation was used to add additional genomes, including draft genomes from NCBI Assemblies and PATRIC. Comparison of the overall microbiome composition between groups was performed by multi-response permutation procedure (MRPP) using the Vegan package in R (114). Microbiome shotgun sequencing reads obtained from littermate HDAC3^FF^ and HDAC3^ΔIEC^ and MHCII^FF^ and MHCII^ΔIEC^ mice were aligned against the cBir1 peptide sequence (115) (AY551005.1) using the tblast command and default settings in the program Diamond (116). Prevalence of this gene is expressed as cBir1 counts per million mapped bacterial reads. For cBir1 quantitative PCR (qPCR) analysis, stool DNA was extracted using the QIAamp Fast DNA Stool Extract Kit (Qiagen) following the manufacturer’s instructions. Bacterial DNA was assessed for cBir1 gene expression by qPCR analysis using the following primer set: cBir1 forward 5′-AAGTACTTTACGGCAGGCGG-3′, cBir1 reverse 5′-TCTGTTCCGTCAGCACCTAC-3′.

### Histological analysis.

Colonic tissue sections were fixed in 4% paraformaldehyde overnight at 4°C, then paraffin-embedded, sectioned, and stained with H&E. For histological scoring, slides were evaluated based on the following parameters: immune cell infiltration, 1–5; mucosal thickening/edema, 1–5; crypt length, 1–5; and crypt abscess/erosion, 1–5.

### Statistics.

Results are shown as mean ± SEM. Tests between 2 groups used a 2-tailed Student’s *t* test. A 1-way ANOVA with Tukey’s test was used for multiple comparisons. Prolapse curves were evaluated using a log-rank Mantel-Cox test. Results were considered significant at *P < 0.05, **P < 0.01, ***P < 0.001, ****P < 0.0001. Statistical significance was calculated using Prism version 7.0 (GraphPad Software).

### Study approval.

All animal studies were approved by the Institutional Animal Care and Use Committee at Cincinnati Children’s Hospital Medical Center.

## Author contributions

EME, SSW, and TA conceptualized the study and designed the study methodology. EME, TYA, VW, TR, BJD, LE, JW, and DBH conducted experiments. EME, TYS, TR, and DBH analyzed data. EME, TA, and SSW wrote, reviewed, and edited the manuscript.

## Supplementary Material

Supplemental data

## Version 1 01/05/2023

In-Press Preview

## Version 2 02/15/2023

Electronic publication
