# Supplementary material for: Intestinal epithelial HDAC3 and MHC class II coordinate microbiota-specific immunity
Source: J Clin Invest. 2023 Feb 15;133(4):e162190. doi: 10.1172/JCI162190 (PMC9927950; doi:10.1172/JCI162190)
Supplement: Supplemental data [file jci-133-162190-s170.pdf]

Supplemental Figure 1

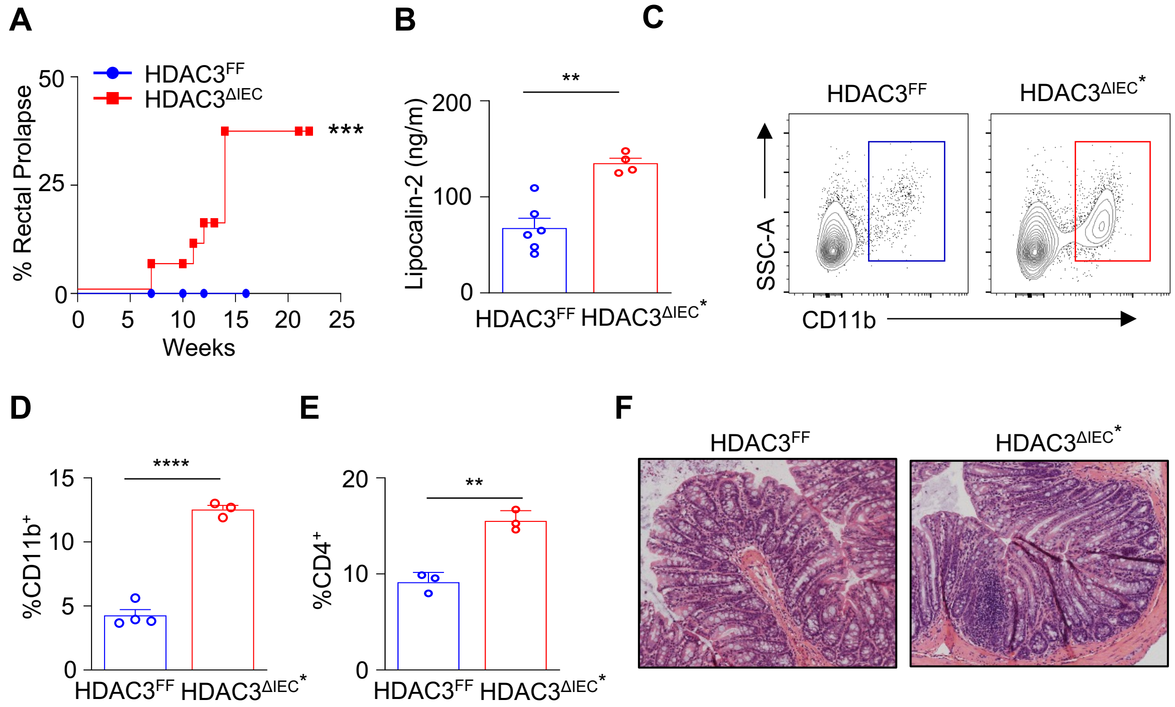

Supplemental Fig. 1. Loss of epithelial HDAC3 expression increases spontaneous intestinal inflammation. (**A**) Frequency of rectal prolapse in HDAC3^{FF} and HDAC3$^{\Delta IEC}$ mice. (**B**) Fecal concentrations of lipocalin-2. (**C, D**) Frequency of CD11b^{+} myeloid cells (Gated Live, CD45^{+}, CD3^{-}) and (**E**) frequency of intestinal CD4^{+} T cells (Gated Live, CD45^{+}) in the large intestine of HDAC3^{FF} and prolapse (*) HDAC3$^{\Delta IEC}$ mice. (**F**) H&E-stained colonic sections. Data represent at least three independent experiments, 3-6 mice per group. **$p<0.01$, ***$p<0.001$, ****$p<0.0001$.

Supplemental Figure 2

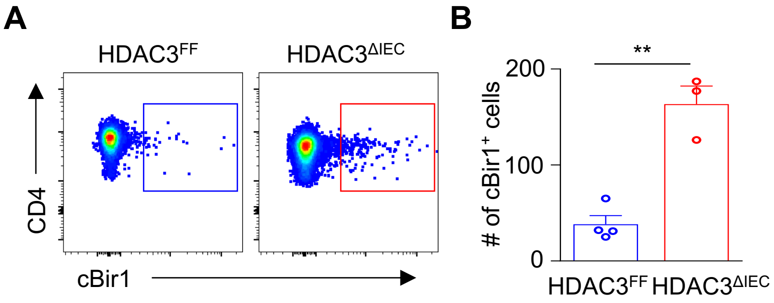

Supplemental Fig. 2. HDAC3 regulates commensal-specific T cells in the small intestine. (**A, B**) Number of cBir1+ CD4 T cells in small intestine of $HDAC3^{FF}$ and $HDAC3^{\Delta IEC}$. $cBir1^{+}$ tetramer cells are gated on live, $CD45^{+}$, lineage (CD11b, B220, Ly6G, CD11c, CD8a)$^{-}$, $CD4^{+}$. Data represent at least three independent experiments, 3-4 mice per group. $^{**}p<0.01$.

Supplemental Figure 3

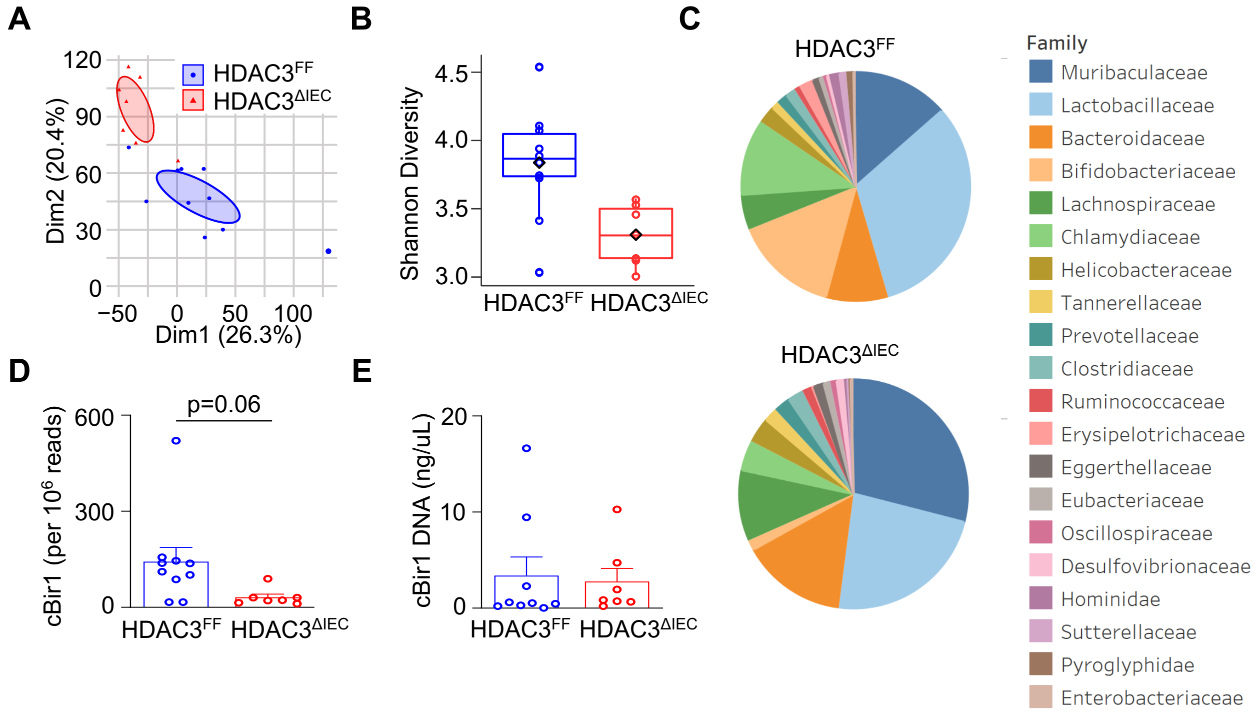

Supplemental Fig. 3. Intestinal microbiota composition in HDAC3^{FF} and HDAC3$^{\Delta IEC}$ mice. (**A**) PCA plot, (**B**) Shannon diversity, and (**C**) bacterial composition in HDAC3^{FF} and HDAC3$^{\Delta IEC}$ mice from metagenomic analyses of stool sample. (**D**) cBir1 counts per million reads and (**E**) cBir1 by qPCR in stool of HDAC3^{FF} and HDAC3$^{\Delta IEC}$ mice.

Supplemental Figure 4

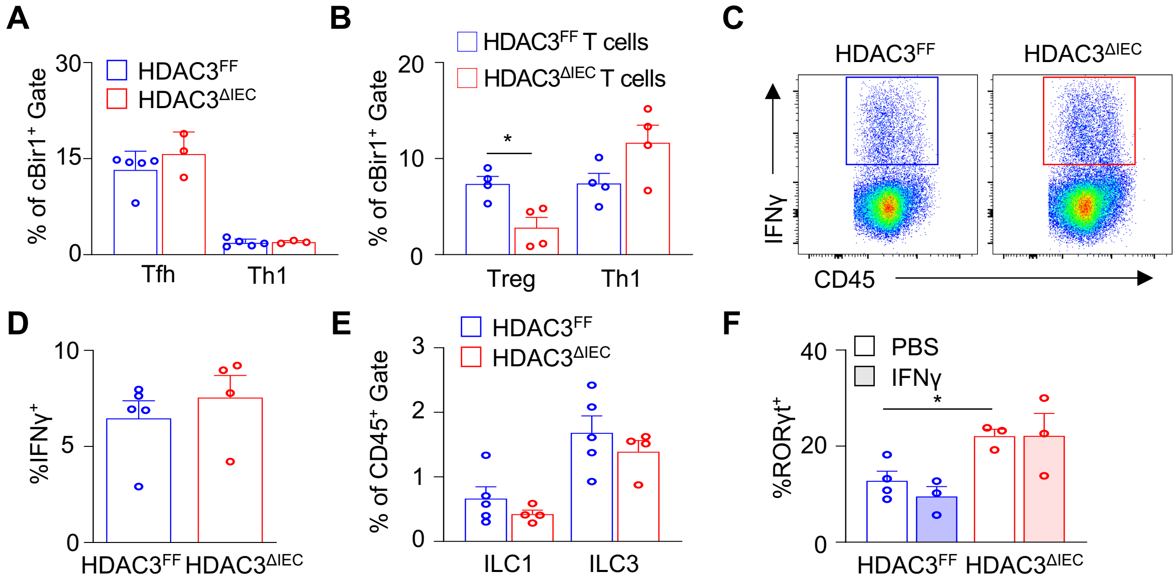

Supplemental Fig. 4. Intestinal immune responses in HDAC3^{ΔIEC} mice. (**A**) Frequency of Th subsets of cBir1$^+$CD4$^+$ T cells from the large intestine of HDAC3^{FF} and HDAC3^{ΔIEC}. (**B**) Frequency of Th subsets of cBir1$^+$ commensal-specific T cells following transfer into Rag1$^{-/-}$ mice. cBir1$^+$ tetramer cells are gated on live, CD45$^+$, lineage (CD11b, B220, Ly6G, CD11c, CD8a)$^-$, CD4$^+$. T follicular (Tfh) cells gated cBir1$^+$, FoxP3$^-$, CXCR5$^+$; Th1 cells gated cBir1$^+$, FoxP3$^-$, CXCR5$^-$, RORγt$^-$, Tbet$^+$. (**C, D**) Frequency of IFNγ$^+$ CD45$^+$ cells and (**E**) frequency of ILC1s and ILC3s in large intestine of HDAC3^{FF} and HDAC3^{ΔIEC} mice at steady state. (**F**) Frequency of RORγt$^+$ cBir1$^+$ cells in intestine of HDAC3^{FF} and HDAC3^{ΔIEC} mice treated with PBS or IFNγ. Data represent at least two independent experiments, 3-5 mice per group. *p<0.05.

Supplemental Figure 5

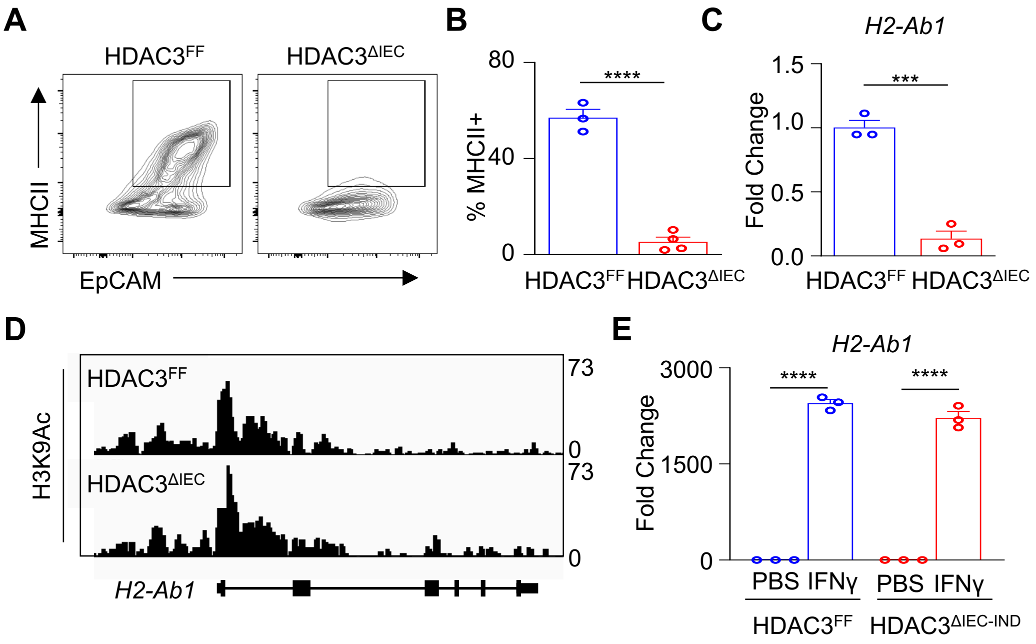

Supplemental Fig. 5. Histone acetylation and IFNγ induction of *H2-Ab1* are not significantly altered in IECs lacking HDAC3. (**A, B**) Frequency of MHCII^{+} IECs from small intestine of HDAC3^{FF} and HDAC3$^{\Delta IEC}$ mice. (**C**) H2-Ab1 mRNA expression in small intestinal IECs from of HDAC3^{FF} and HDAC3$^{\Delta IEC}$ mice. (**D**) ChIP-seq for H3K9Ac in primary IECs isolated from the large intestine. (**E**) H2-Ab1 mRNA expression in HDAC3^{FF} and HDAC3$^{\Delta IEC\text{-}IND}$ organoids treated with PBS or IFNγ. Data are representative of at least two independent experiments, 3-4 mice per group. ***p<0.001, ****p<0.0001.

Supplemental Figure 6

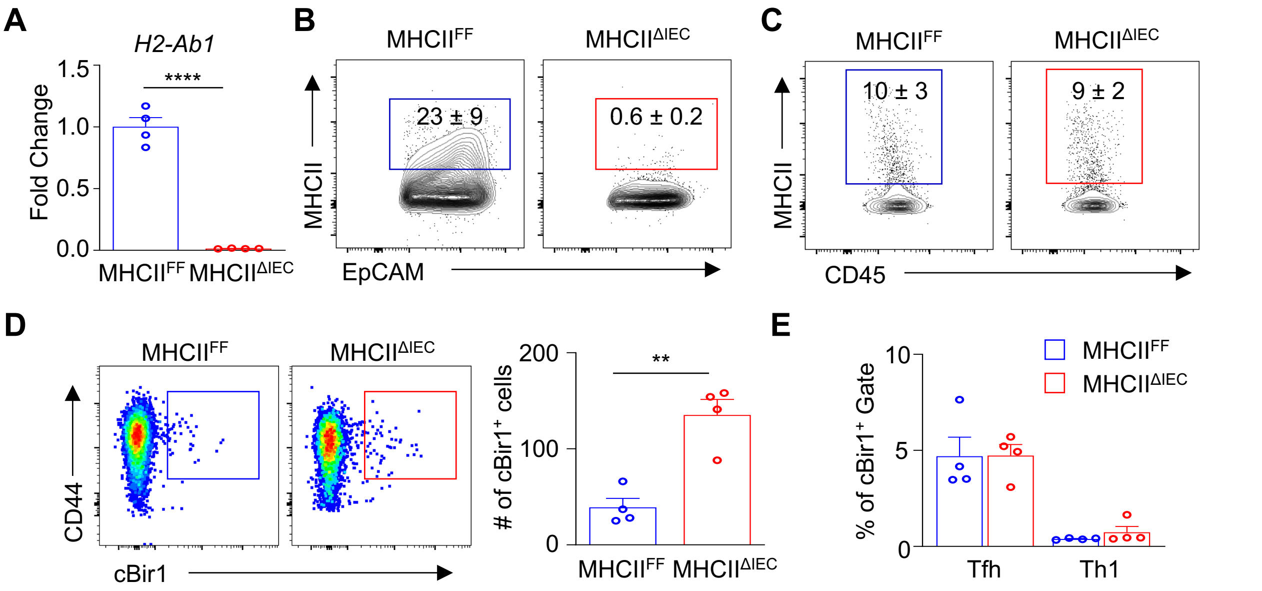

Supplemental Fig. 6. Confirmation of IEC-specific depletion of MHCII. (**A**) H2-Ab1 mRNA expression in IECs isolated from the large intestine of $MHCII^{FF}$ and $MHCII^{\Delta IEC}$ mice. (**B**) Surface MHCII expression in $EpCAM^{+}$, $CD45^{-}$ cells from $MHCII^{FF}$ and $MHCII^{\Delta IEC}$ mice. (**C**) Surface MHCII expression in $EpCAM^{-}$, $CD45^{+}$ cells from $MHCII^{FF}$ and $MHCII^{\Delta IEC}$ mice. (**D**) Number of $cBir1^{+}$ $CD4^{+}$ T cells in small intestine of $MHCII^{FF}$ and $MHCII^{\Delta IEC}$ mice. (**E**) Frequency of Th subsets of $cBir1^{+}CD4^{+}$ T cells from colon of $MHCII^{FF}$ and $MHCII^{\Delta IEC}$ mice. $cBir1^{+}$ tetramer cells are gated on live, $CD45^{+}$, lineage (CD11b, B220, Ly6G, CD11c, CD8a)$^{-}$, $CD4^{+}$. T follicular (Tfh) cells gated $cBir1^{+}$, $FoxP3^{-}$, $CXCR5^{+}$; Th1 cells gated $cBir1^{+}$, $FoxP3^{-}$, $CXCR5^{-}$, $ROR\gamma t^{-}$, $Tbet^{+}$. Data represent at least three independent experiments, 3-4 mice per group. **$p<0.01$, ****$p<0.0001$

Supplemental Figure 7

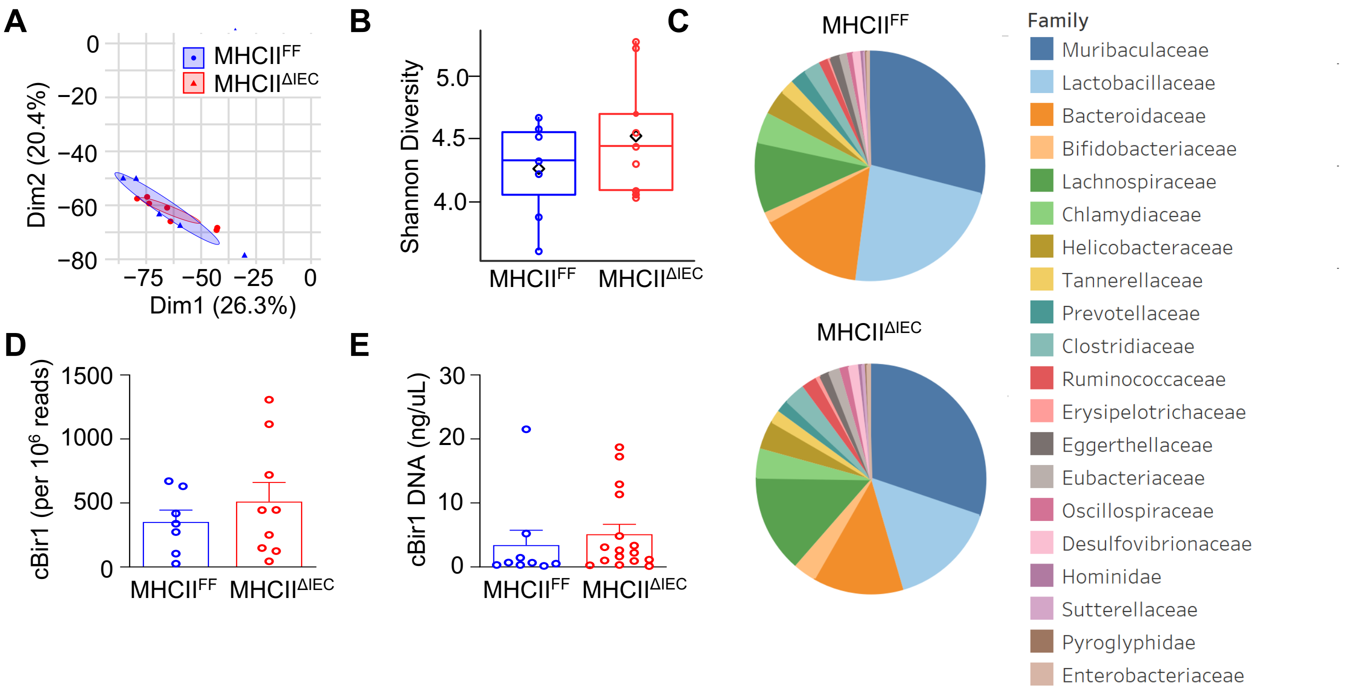

Supplemental Fig. 7. Intestinal microbiota composition in MHCII^{FF} and MHCII$^{\Delta IEC}$ mice. (**A**) PCA plot, (**B**) Shannon diversity, and (**C**) bacterial composition in MHCII^{FF} and MHCII$^{\Delta IEC}$ mice from metagenomic analyses of stool sample. (**D**) cBir1 counts per million reads and (**E**) cBir1 by qPCR in stool of in MHCII^{FF} and MHCII$^{\Delta IEC}$ mice.

Supplemental Figure 8

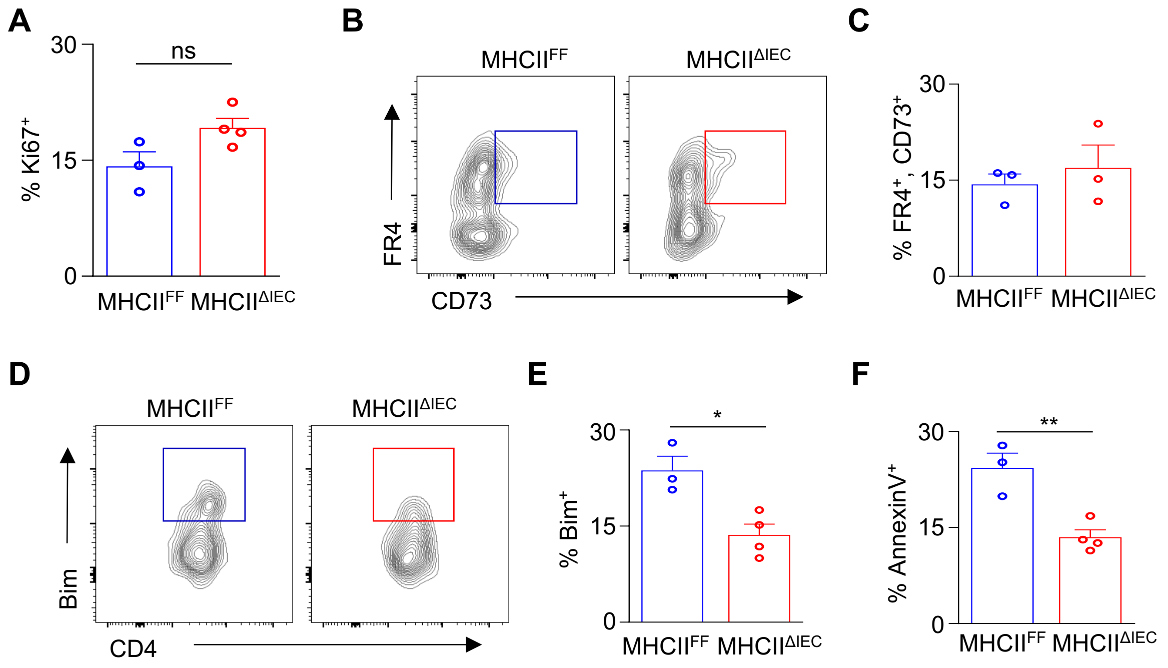

Supplemental Fig. 8. Commensal-specific $CD4^+$ T cells from $MHCII^{\Delta IEC}$ mice exhibit reduced markers of apoptosis. (**A**) Frequency of $Ki67^+$, (**B**, **C**) $FR4^+$, $CD73^+$, (**D**, **E**) Bim^+ in $cBir1^+$ $CD4^+$ T cells from the large intestine of $MHCII^{FF}$ and $MHCII^{\Delta IEC}$ mice. (**F**) Frequency of $AnnexinV^+$ antigen experienced intestinal $CD4^+$ T cells from the large intestine of $MHCII^{FF}$ and $MHCII^{\Delta IEC}$ mice. Data represent at least two independent experiments, 3-4 mice per group. *p<0.05, **p<0.01, ns=not significant.

Supplemental Figure 9

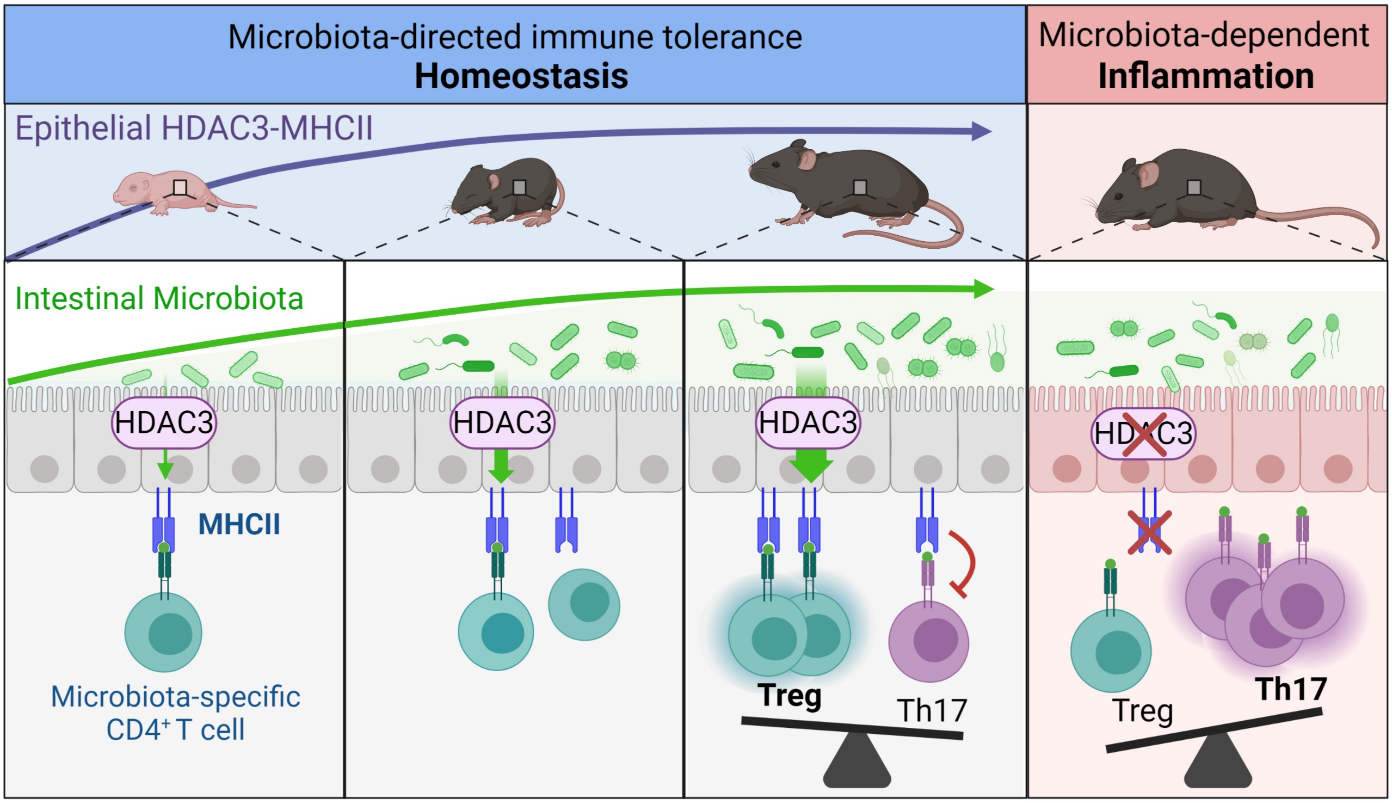

Supplemental Fig. 9. Intestinal epithelial MHC class II regulation by HDAC3 instructs microbiota-specific CD4⁺ T cells. HDAC3 regulation of epithelial MHCII is activated early and concurrently with microbiota colonization. This pathway is necessary to control local accumulation of T cells that respond to commensal microbes later in life. Loss of intestinal epithelial HDAC3 results in reduced commensal-specific Tregs and an increase in commensal-specific Th17 cells. Thus, microbiota drive tolerance in the intestine by inducing an epithelial mechanism that directs local CD4⁺ T cell subsets that recognize commensal antigens and decrease susceptibility to microbiota-sensitive inflammation. Created with Biorender.com.
